# Incidence of hypernatremia in dogs treated with single dose activated charcoal for acute toxicant ingestion: multi-center retrospective study (2018–2023)

**DOI:** 10.3389/fvets.2025.1547076

**Published:** 2025-02-26

**Authors:** Timothy Young, Rebecca A. L. Walton, Poyee Cheng, Jiazhang Cai, Jonathan P. Mochel, Katherine Peterson

**Affiliations:** ^1^Veterinary Centers of America (VCA) West Los Angeles, Los Angeles, CA, United States; ^2^Precision One Health Initiative, The University of Georgia, Athens, GA, United States; ^3^BluePearl, Duluth, MN, United States

**Keywords:** decontamination, electrolyte derangements, toxicity, sorbitol, hypernatremia

## Abstract

**Objective:**

To retrospectively evaluate the incidence and clinical significance of hypernatremia in dogs administered a single dose of activated charcoal (AC) or activated charcoal with sorbitol (ACS) for acute toxicant ingestion.

**Methods:**

Retrospective study between the years 2018–2023. Ninety-six dogs evaluated by a university teaching hospital and private practice emergency hospital treated for acute toxicant ingestion with a single dose of activated charcoal, with or without sorbitol.

**Results:**

Medical records were retrospectively reviewed. No dog developed hypernatremia, defined as sodium >155 mEq/L, during the study period. The toxicant ingested was not significantly associated with a change in sodium (Na) at any time point (*P* = 0.433 at 6–12 h, *P* = 0.09 at 12–14 h, and *P* = 0.486 at 24–48 h). Ingestion of multiple toxicants, compared to single toxicant ingestion, was also not significantly associated with a change in Na at any time point (*P* = 0.126 at *P* = 6–12 h, *P* = 0.452 at 12–24 h, and *P* = 0.516 at 24–48 h). Time from ingestion to presentation was not significantly associated with a change in Na at any time point (*P* = 0.422 at 6–12 h, *P* = 0.881 at 12–24 h, and *P* = 0.516 at 24–48 h). The administration of AC compared to ACS was not significantly associated with a change in Na at any time period (*P* = 0.715 at 6–12 h, *P* = 0.137 at 12–24 h, *P* = 0.582 at 24–48 h). There was a significant difference between Na on presentation and at 12–24 h and 24–48 h (*P* = 0.025 and *P* = 0.015, respectively.) Median Na values decreased at all-time points when compared to presentation, −0.90 at 6–12 h (standard deviation (SD) 2.74), −1.38 at 12–24 h (SD 4.81) and −2.11 at 24–48 h (SD 5.34).

**Conclusions:**

This study demonstrated a statistically significant, but unlikely clinically significant, decrease in Na in dogs who received single dose AC or ACS for acute toxicant ingestion.

## 1 Introduction

Activated charcoal (AC) is an adsorbent used for detoxification treatment in veterinary medicine (1, 2). Activated charcoal may increase the clearance of certain toxicants via binding of the toxicant within the gastrointestinal tract, which reduces absorption and disrupts enterohepatic recirculation of a poison (1–3). In humans, AC may decrease the absorption of certain toxicants by up to 52% if administered 30 min after ingestion (3). *In vitro* studies have demonstrated high efficacy of AC with a 99.6% reduction in acetaminophen concentrations and reduction of maximum plasma concentrations and pharmacokinetic area under the curve of carprofen (4, 5). Sorbitol is commonly added to AC (ACS) as a cathartic to aid in the expulsion of toxicants and to decrease gastrointestinal transit time, resulting in reduced toxicant absorption and improved decontamination (1, 2, 5).

Despite the reported benefits of AC/ACS, there are a lack of veterinary studies assessing potential adverse effects. In humans, adverse effects associated with AC/ACS are uncommon and may include electrolyte derangements, gastrointestinal obstruction, or aspiration of AC/ACS (3). Development of electrolyte abnormalities, including hypernatremia is suspected to be due to the osmotic effects of AC or ACS, resulting in free water loss into the gastrointestinal tract (6). While electrolyte abnormalities are atypical, they are reported with just a single dose AC/ACS (7). Other complications associated with AC/ACS in dogs include alterations in serum osmolality and lactate, and case reports of gastrointestinal obstruction secondary to AC granule impaction and incidents of aspiration pneumonia have been reported (8–10). A prospective, cross-over study in nine healthy dogs administered a single dose of 2 g/kg ACS revealed a statistically significant increase in serum sodium (Na), with two dogs developing hypernatremia, defined as serum Na levels up 155 mEq/L (11). One dog developed muscle tremors and paraparesis associated with the hypernatremia, which resolved with supportive care (11). It is important to note that both groups of dogs in this study were clinically healthy and had water withheld for 12 h following administration of ACS (11). These dogs were also administered 2 g/kg, which is on the high end of recommended oral AC dosing, which resulted in significant weight loss (11). While the hypernatremia was clinically significant in this study, the clinical signs associated with hypernatremia in one dog were minimal and no long-term complications were reported (11).

Concern for hypernatremia may impact decision making regarding AC/ACS administration, despite its noted beneficial effects on toxicant absorption and elimination. Currently, no clinical studies exist regarding the impact of AC/ACS on electrolyte status. The goal of this study was to evaluate the incidence and clinical relevance of hypernatremia after administration of single-dose AC/ACS in dogs treated for acute toxicant ingestion.

## 2 Materials and methods

### 2.1 Case selection

The computerized medical record system at a large urban specialty hospital and a university veterinary teaching hospital were searched for dogs administered activated charcoal for toxicant ingestion between February 2018 and August 2023. Search criteria involved financial codes for “activated charcoal” and “activated charcoal with sorbitol.” Dogs were included if a single dose of activated charcoal was administered due to known toxicant ingestion and a Na level was evaluated both prior to AC/ACS (Toxiban, Lloyd Inc, Shenandoah, IA) administration and within 6–48 h after AC/ACS administration. Single dose AC/ACS was selected based on experimental studies and the impact of single dose AC on serum Na in dogs (11). Dogs were excluded if they received AC/ACS for reasons other than known toxicant ingestion, based on history taking from owner, received more than one dose of AC/ACS or if Na levels were not evaluated before or after charcoal administration. The following data was collected: toxicant ingested, time from ingestion to presentation, induction and success of emesis with the administration of apomorphine, dose of AC/ACS administered, and point-of-care (POC) blood work prior to administration of AC/ACS. Point-of-care bloodwork included Na, chloride (Cl), packed cell volume (PCV), total plasma protein (TPP), blood glucose (BG) and lactate. Any repeat POC blood work at 6–12 h, 12–24 h and at 24–48 h after AC/ACS administration was also considered where available. Additional treatment, including outpatient therapy or hospitalization was evaluated, along with the administration of IV fluids including fluid type and fluid rate, and survival to discharge.

### 2.2 Statistical analysis

The aim of this study was to determine the incidence of hypernatremia in dogs treated with a single dose of AC. Sodium levels were measured for each dog at four time points: upon presentation, 6–12, 12–24, and 24–48 h after AC administration. Initially, sodium concentrations at each time point were treated as single distributions. A two-tailed Wilcoxon rank-sum test was used to assess whether the distribution of sodium concentrations changed significantly before and after AC administration over the specified time periods.

Additional analyses were conducted to evaluate factors associated with changes in sodium concentrations. For categorical variables with more than two categories, the Kruskal-Wallis rank-sum test was used to assess differences in sodium concentration changes across categories. For continuous variables, Pearson's product-moment correlation test was applied to examine the strength of correlation between these variables and sodium concentration changes.

In this study, *P*-values < 0.05 were considered statistically significant. All analyses were performed using R software. Data visualizations were generated using the ggplot2 package in R version 4.3.1 (12).

## 3 Results

A total of 472 dogs that received AC or ACS were identified through the medical records database. Two hundred and seventy-nine dogs were excluded due to lack of point of care blood work including Na values following AC/ACS administration, incomplete medical records, or administration of AC/ACS due to non-toxic treatment. Ninety-seven dogs were excluded due to receiving multi-dose AC and were evaluated as a separate population. In total, 96 dogs met the inclusion criteria for this study. Thirty-seven dogs were spayed females, 35 castrated males, 13 intact females, and 11 intact males. The median age was 3 years (range 0.2–14 years). The median weight was 14.3 kg (range 1.8–43.5 kg). The most commonly reported breeds were mixed breed dog (44), Labrador retriever (six), German shepherd (four), and French bulldog (four), with other breeds represented by fewer than 4 dogs.

Eighty-two (85.4%) dogs ingested a single toxicant. Of the dogs that ingested a single toxicant, 28 dogs (34%) ingested grapes/raisins, 24 dogs (29%) ingested non-steroidal anti-inflammatory drugs, nine dogs (11%) ingested rodenticide (five cholecalciferol, three bromethalin and one unknown), seven dogs (8.5%) ingested chocolate, three dogs (5%) ingested vitamin D supplements, three dogs (3.6%) ingested calcium channel blockers, two dogs (2.4%) ingested Sago palms, and 1 dog ingested each of the following toxicants: duloxetine, mushrooms, primidone, xylitol, levodopa, and phenylephrine. Fourteen dogs (14.6%) ingested multiple toxicants. Six dogs ingested chocolate and grapes/raisins, two dogs ingested vitamin D supplements and a selective serotonin reuptake inhibitor, one dog ingested chocolate and marijuana, one dog ingested acetaminophen and aspirin, one dog ingested ibuprofen and acetaminophen, one dog ingested xylitol, raisins, and chocolate and one dog ingested xylitol and melatonin. The type of toxicant ingested was not significantly associated with a change in Na at any time point with a confidence level of 0.95 (*P* = 0.433 at 6–12 h, *P* = 0.09 at 12–14 h, and *P* = 0.486 at 24–48 h; Table 1) Ingestion of multiple toxicants, compared to a single toxicant, was also not significantly associated with the change in Na at any time point (*P* = 0.126 at 6–12 h, *P* = 0.452 at 12–24 h, and *P* = 0.516 at 24–48 h). Nineteen dogs presented < 1 h post ingestion, 52 dogs within 1–6 h of ingestion, two within 6–12 h of ingestion, four >12 h, and for 19 dogs the timing was unknown. The time from ingestion to presentation was not significantly associated with change in Na at any time point (*P* = 0.422 at 6–12 h, *P* = 0.881 at 12–24 h, and *P* = 0.516 at 24–48 h). Twenty-one dogs vomited prior to presentation and 78 dogs had emesis induced at the time of presentation, which was successful in 77 (98.7%) dogs. Eleven dogs did not vomit or have emesis induced, suspected to be due to the timing of ingestion. The induction of emesis was not significantly associated with change in Na at any time point (*P* = 0.747 at 6–12 h, *P* = 0.248 at 12–24 h and *P* = 0.826 at 24–48 h). The majority of patients, 79 dogs (81%) received ACS, while 18 dogs (19%) received AC. The administration of AC compared to ACS was not significantly associated with a change in Na at any time period (*P* = 0.715 at 6–12 h, *P* = 0.137 at 12–24 h, *P* = 0.582 at 24–48 h). The median dose of activated charcoal given was 1 g/kg (range 0.5–2 g/kg). Eighty dogs (83%) were hospitalized, and 16 dogs (17%) were treated on an outpatient basis. The median duration of hospitalization was 38 h (range 4–312 h). Seventy-six dogs were hospitalized on intravenous fluids, 72 dogs were hospitalized on Lactated Ringers solution, two dogs were hospitalized on Normosol-R, and two dogs were hospitalized on 0.45% saline, neither of which were hypernatremic at the time of fluid initiation, and 3 dogs did not receive intravenous fluids during hospitalization. The median fluid rate was 90 ml/kg/day (range 38–182 ml/kg/day). Hospitalization, intravenous fluids administration, and the type of intravenous fluids were not significantly associated with change in Na at any time point. All dogs survived to discharge, regardless of inpatient vs. outpatient care. No dogs were noted to have any concerns for hypernatremia noted in the medical record.

**Table 1:** In 96 dogs given a single dose of activated charcoal (AC) or activated charcoal with sorbitol (ACS), the association between toxicant and treatment variables and sodium (Na) values at 6–12 h following initial presentation, 12–24 and 24–48 h.

	**Na @ 6–12 h**	**Na @ 12–24 h**	**Na @ 24–48 h**
Type of toxicant	*P* = 0.433	*P* = 0.09	*P* = 0.486
Ingestion of multiple toxicants	*P* = 0.126	*P* = 0.452	*P* = 0.516
Time from ingestion to presentation	*P* = 0.422	*P* = 0.881	*P* = 0.516
Induction of emesis	*P* = 0.747	*P* = 0.248	*P* = 0.826
Administration of AC compared to ACS	*P =* 0.715	*P* = 0.137	*P* = 0.582
Hospitalization compared to outpatient	*P =* 0.927	*P =* 0.299	*P =* 0.177
Fluid type	*P =* 0.482	*P =* 0.694	*P =* 0.653
Fluid rate	*P =* 0.142	*P =* 0.864	*P =* 0.362

Point-of-care blood work for all dogs is presented in Table 2. Two dogs were noted to be hypernatremic at the time of presentation, prior to administration of AC/ACS, however, no dogs were hypernatremic at any time point following AC/ACS administration. No POC value was associated with a change in Na at any time point. There was a significant difference between Na on presentation and at 12–24 h or 24–48 h (*P* = 0.025 and *P* = 0.015, respectively), however, not at 6–12 h (*P* =0.439). Median Na values decreased at all-time points, compared to presentation, −0.90 mEq/L at 6–12 h [standard deviation (SD) 2.74 mEq/L], −1.38 mEq/L at 12–24 h (SD 4.81 mEq/L) and −2.11 mEq/L at 24–48 h (SD 5.34 mEq/L).

**Table 2:** Point-of-care bloodwork at all-time points in 96 dogs given a single dose of activated charcoal or activated charcoal with sorbitol following a toxicant ingestion.

	**Normal range**	**Presentation**	**6–12 h**	**12–24 h**	**24–48 h**
Na (mEq/L)	141–155	(*n* = 96) 146.2 (137–162)	(*n* = 21) 146.2 (136.4–154.6)	(*n* = 74) 145 (132–153)	(*n* = 39) 145 (136–154)
Cl (mEq/L)	109–121	(*n* = 84) 112 (102–126)	(*n* = 21) 112.6 (104.8–118.1)	(*n* = 68) 113 (106–129)	(*n* = 37) 112.3 (107–128)
PCV (%)	37–55	(*n* = 88) 48 (28–69)	(*n* = 24) 45 (34–59)	(*n* = 34) 44 (33–60)	(*n* = 14) 43 (32–454)
TPP (g/dl; g/L)	5.4–7.5; 54–75	(*n* = 84) 7; 70 (5.2–10; 52–100)	(*n* = 22) 6.8; 68 (5.4–8.6; 54–86)	(*n* = 35) 6.4; 64 (4.9–9; 49–90)	(*n* = 12) 6.3; 63 (4.3–7.8; 43–78)
BG (mg/dl; mmol/L)	81–118; 4.5–6.6	(*n* = 90) 105; 5.8 (47–236; 2.6–13.1)	(*n* = 24) 103; 5.7 (69–129; 3.8–7.2)	(*n* = 43) 101; 5.6 (72–149; 4.0–8.3)	(*n* = 24) 109; 6.1 (76–133; 4.2–7.4)
Lactate (mmol/L; mg/dl)	0.40–2.80; 3.6–25.2	(*n* = 64) 2.3; 20.7 (0.3–6.6; 2.7–59.5)	(*n* = 24) 1.9; 17.1 (0.7–4.7; 6.3–42.3)	(*n* = 29) 1.3; 11.7 (0.5–3.1; 4.5–27.9)	(*n* = 10) 1.2; 10.8 (0.4–3.4; 3.6–30.6)

N, number of dogs, followed by median and range.

The majority, 76 dogs (79%), were hospitalized on intravenous fluids and no dogs were water restricted. Of the dogs that were not hospitalized on intravenous fluids, median Na on presentation was 146.2 mEq/L (range 137–163 mEq/L). The median Na at 6–12 h was 146.1 mEq/L (range 136.4–154.6 mEq/L), 145 mEq/L (range 132–153 mEq/L) at 12–24 h and 145 mEq/L (range 136–154 mEq/L) at 24–48 h. No significant difference in serum Na levels between fluid rate or type was found at any time points (*P* = 0.42 and 0.12, respectively).

## 4 Discussion

This retrospective study aimed to evaluate hypernatremia as a potential complication in dogs receiving a single dose of AC/ACS for acute toxicant ingestion. The results demonstrated a statistically significant, but not clinically relevant, decrease in Na concentrations at 12–24 and 24–48 h, compared to presenting Na. No dogs developed hypernatremia in this retrospective study.

Activated charcoal, with or without sorbitol, is commonly used due to its adsorbent properties and there is concern for development of hypernatremia in veterinary patients after administration, with or without sorbitol (1, 2). In people, AC/ACS has been associated with severe hypernatremia, more prevalent in pediatrics (13, 14). In dogs there is little information to date, however, a cross-over experimental study in 9 healthy dogs receiving a single dose of 2 g/kg of AC demonstrated a statistically significant increase in serum Na following AC administration in all dogs, with a median serum Na of 151.1 mEq/L at 6 h post-treatment (11). The previous study is in contrast to the current study that noted a statistically, but not clinically, significant decrease in Na levels and no evidence of hypernatremia in any dogs. In the present study, no dogs were noted to have serum Na levels above reference range (> 155 mEq/L) at any time period following administration of AC/ACS (11). It is important to note that the previous study was an experimental study where healthy dogs were administered larger doses of activated charcoal than the median dose administered to the clinical patients in our study. While the range of activated charcoal doses listed in texts is from 1 to 4g/kg, the lower dose of 1 g/kg is commonly recommended and may reduce the risk of adverse electrolyte and other biochemical changes (1, 2). Additionally, the dogs in the previous study also had drinking water withheld and were not administered any fluid therapy (11). In contrast, the present study provided free access to water all cases and most dogs also received intravenous fluids. The majority of dogs in the present study were maintained with intravenous fluids but receiving fluid therapy, fluid type, and fluid rate were not significantly associated with changes in Na at any time point. This is likely explained by the fact that Na concentrations and osmolality are strictly maintained by homeostatic mechanisms and a change of 1–2 mOsm/L sensed by the hypothalamus induces compensatory mechanisms such a ADH release and thirst response (13). There appears to be low risk of developing hypernatremia in dogs treated with single dose AC/ACS if either maintained on intravenous fluids or allowed free access to water but further studies are needed to confirm these results.

Point-of-care blood work is commonly performed in acute toxicities and abnormalities may exist depending on the toxicant ingested or secondary to clinical signs resulting ingestion. The association between POC blood work and changes in Na following AC/ACS administration were evaluated in the present study and no association between POC blood work and change in Na was noted. This may be explained by the fact that the majority of dogs in this study received IV fluids to aid in hydration with additional free water access or may be due to the clinical status of patients in this study. Additionally, the median PCV and TPP were within normal limits, indicating that clinically significant dehydration was unlikely be to present in most dogs.

There are several limitations to this study. As a retrospective study, treatments such as IV fluid rates, fluid type, and dose and timing of AC administered were not controlled, which may have impacted Na levels. Additionally, water intake in the form of drinking was not quantified for any dogs, including those treated as inpatients and outpatients. The majority of dogs in this study were hospitalized, with only 17% of dogs being treated on an outpatient basis. Standardized treatments could be considered for future evaluation. Sodium values were also measured at varied time frames, which may have affected the results. While the toxicant did not appear to have an effect on the development of hypernatremia in this study, multiple toxicants were represented, many of them in low numbers, and including multiple toxicant ingestions were included. The dose of AC/ACS also varied, which may have affected outcome.

In conclusion, the administration of a single dose of AC/ACS in dogs with acute toxicant ingestion was not associated with the development of hypernatremia in this retrospective study.

## Data Availability

The original contributions presented in the study are included in the article/supplementary material, further inquiries can be directed to the corresponding author.
